# Differential Effects of ‘Vaping’ on Lipid and Glucose Profiles and Liver Metabolic Markers in Obese Versus Non-obese Mice

**DOI:** 10.3389/fphys.2021.755124

**Published:** 2021-11-04

**Authors:** Hui Chen, Gerard Li, Yik Lung Chan, Hui Emma Zhang, Mark D. Gorrell, Carol A. Pollock, Sonia Saad, Brian G. Oliver

**Affiliations:** ^1^School of Life Sciences, Faculty of Science, University of Technology Sydney, Sydney, NSW, Australia; ^2^Centenary Institute, Faculty of Medicine and Health, The University of Sydney, Sydney, NSW, Australia; ^3^Renal Research Laboratory, Kolling Institute of Medical Research, The University of Sydney, Sydney, NSW, Australia; ^4^Respiratory Cellular and Molecular Biology, Woolcock Institute of Medical Research, The University of Sydney, Sydney, NSW, Australia

**Keywords:** liver, abdominal obesity, free fatty acid, triglycerides, glucose tolerance

## Abstract

Tobacco smoking increases the risk of metabolic disorders due to the combination of harmful chemicals, whereas pure nicotine can improve glucose tolerance. E-cigarette vapour contains nicotine and some of the harmful chemicals found in cigarette smoke at lower levels. To investigate how e-vapour affects metabolic profiles, male Balb/c mice were exposed to a high-fat diet (HFD, 43% fat, 20kJ/g) for 16weeks, and e-vapour in the last 6weeks. HFD alone doubled fat mass and caused dyslipidaemia and glucose intolerance. E-vapour reduced fat mass in HFD-fed mice; only nicotine-containing e-vapour improved glucose tolerance. In chow-fed mice, e-vapour increased lipid content in both blood and liver. Changes in liver metabolic markers may be adaptive responses rather than causal. Future studies can investigate how e-vapour differentially affects metabolic profiles with different diets.

## Introduction

In recent years, e-cigarettes have gained significant popularity among younger people, where they are considered as a must-have electronic and a ‘safe cigarette’. Although e-cigarettes were originally marketed as a smoking cessation aid to reduce tobacco use, they are largely used recreationally by those who never used tobacco cigarettes, despite the latest reports of acute lung injury and death in the United States (Layden et al., 2019).

The negative impacts of tobacco smoking on metabolic disorders in adults and adolescents have been well documented (Oh et al., 2005; Weitzman et al., 2005). Nicotine alone (i.e. as a free chemical) can positively increase glucose uptake into tissues after chronic administration in both lean and obese rodents (Liu et al., 2003; Vu et al., 2014). The effects of tobacco smoke and pure nicotine can be different, as tobacco smoke contains more than 5,000 chemicals resulting in toxicity to the body, including increased risk of metabolic diseases and cancer. Nicotine, although addictive, is also anti-inflammatory (Kalra et al., 2004; Cohen et al., 2007). This leads to the discrepancies between *in vivo* studies using pure nicotine and direct cigarette smoke. E-cigarette vapour, which is claimed to be water and nicotine, is clearly not so innoxious as it contains various harmful chemicals which are also found in cigarettes (Li et al., 2021) that may potentially affect lipid and glucose metabolism. Due to the relatively recent emergence of e-cigarettes, there is limited research on the impact of e-cigarette use beyond the respiratory system, which is also dominated by studies funded by the tobacco industry (Pisinger and Døssing, 2014). Human studies in this area are difficult and are affected by various confounders, such as dietary habits, pre-existing health conditions and the dual use of e-cigarettes and tobacco cigarettes (Kim et al., 2020). E-cigarette users have been reported to have a larger body mass index, and a large proportion of dual users have abdominal obesity (Lanza et al., 2017; Górna et al., 2020; Kim et al., 2020). Thus, it is not surprising to find a higher risk of metabolic syndromes in this population, such as an increased blood glucose level or a prediabetic status (Lanza et al., 2017; Górna et al., 2020; Kim et al., 2020). Long-term longitudinal studies on e-cigarette users are not yet available because of their relatively recent entry into the market. Therefore, it is unclear whether the metabolic disorders in e-cigarette users are due to a natural progression due to pre-existing conditions/unhealthy lifestyles, e-cigarette use or the combination of both.

Obesity causes low grade systemic inflammation, due to the recruitment and accumulation of tissue macrophages in response to lipid influx (Lumeng et al., 2008). This is believed to be the dominant mechanism for the development of insulin resistance and liver steatosis during obesity. In the ApoE^−/−^ mouse, in which liver steatosis readily develops, the combination of e-cigarette aerosol and high-fat diet (HFD, 60% fat) increased liver triglyceride deposition, oxidative stress, and DNA damage (Espinoza-Derout et al., 2019; Hasan et al., 2019). This was suggested to be due to increased lipolysis in the fat tissue, which increases plasma free fatty acid (FFA) levels, resulting in increased ectopic lipid synthesis and deposition in the liver (Espinoza-Derout et al., 2019). However, the dose of nicotine in this study was very high (equivalent to heavy smokers, i.e. 18 cigarettes/day). Additionally, conclusions from genetically modified mice may only apply to those with severe, pre-existing metabolic disorders. A recent review indicated that the evidence is inconclusive on the risk of type 2 diabetes in e-cigarette users and that current animal studies cannot provide an answer, either due to issues with experimental design (e.g. the use of non-vaporised e-fluids or pure nicotine) or the difficulties of eliminating confounders (e.g. duel use of tobacco cigarette and e-cigarette or pre-existing conditions prior to the use of e-cigarette; Górna et al., 2020). Therefore, a well-controlled animal study using wild-type rodent strains is necessary.

The manufacture and sale of e-fluids are not well regulated. For example, in New York, only the sale of tobacco-flavoured e-fluids is permitted to reduce the potential harm induced by the flavouring chemicals. However, in Australia, the sale of e-fluid is permitted without restrictions on flavour, but nicotine is not a legally allowed additive. While nicotine is the accepted addictive substance in the e-fluid, other additives (e.g. pyrazines) have been found in nicotine-free e-fluid, which alone can cause addiction (Alpert et al., 2015). We previously also found that exposure to nicotine-free e-cigarette vapour can increase lung inflammatory responses and impair memory in mice (Chen et al., 2018a, 2020). However, its impact on glucose and lipid metabolism is unclear. Therefore, using our well-established HFD diet feeding protocol (Glastras et al., 2016; Chen et al., 2018b; Komalla et al., 2020), we aimed to investigate in a mouse model whether nicotine-containing or nicotine-free e-vapour inhalation interacts with diet to affect glucose tolerance, blood lipid levels and liver metabolic markers.

## Materials and Methods

### Animal Experiments

The experiments were approved by the Animal Ethics and Care Committee at Northern Sydney Local Health District (RESP17/93) and all experiments were performed according to the Australian National Health & Medical Research Council Guide for the Care and Use of Laboratory Animals. Balb/c mice were used due to their susceptibility to cigarette smoke-induced lung pathology and systemic inflammatory and fibrotic responses, as well as our previously published protocol (Vlahos et al., 2006; Chen et al., 2020). The experimental protocol in the same animals has been published in detail previously (Chen et al., 2020). Briefly, male Balb/c mice (7weeks, average body weight 20.3g) were housed in individually ventilated cages (3–5/cage) with Pura paper premium bedding and fed a commercially available pellet HFD (43% energy from fat, 20kJ/g, Cat# SF03-20, Specialty Feeds, WA, Australia) for 16weeks to induce obesity, with standard rodent chow as control (14% energy from fat, 14kJ/g, Gordon’s Specialty Stockfeeds, NSW, Australia). From weeks 11–16, two sub-groups of mice in each dietary group were exposed to nicotine-containing [e-cig18, 18mg/ml (regular strength), 50% Propylene Glycol (PG)/50%Vegetable Glycerin (VG), tobacco flavour and Vaper Empire, VIC] and nicotine-free e-vapour [e-cig0; 50% Propylene Glycol (PG)/50%Vegetable Glycerin (VG), tobacco flavour and Vaper Empire, VIC] in a 19L chamber for 30min, twice daily for 6weeks (Chen et al., 2020). The mice with sham exposure were exposed to room air in the identical chamber in the fume hood. Blood cotinine levels measured the following morning after the last e-vapour exposure in these mice were published previously to confirm nicotine exposure (Chen et al., 2020). This generated six experimental groups (*n*=15), Chow+sham, Chow+e-cig18, Chow+e-cig0, HFD+sham, HFD+e-cig18 and HFD+e-cig0. The 24h caloric intake was measured once every 2weeks by the weight difference of the pellets on the cage lid within 24h. Any residue pellets on bedding were also included. The average of caloric intake in the weeks 12, 14 and 16weeks is reported here. At 15weeks of feeding, an intraperitoneal glucose tolerance test was performed as previously described (Chen et al., 2014). After 5h of fasting, the mice were challenged by glucose injection (2g/kg, ip) and blood glucose levels were measured at 0, 15, 30, 60 and 90min. The area under the curve (AUC) of the blood glucose curve was calculated for each mouse. At the endpoint, mice were fasted overnight. After the induction of deep anaesthesia (2% isoflurane), blood was collected *via* cardiac puncture, and the plasma was stored at −20°C for further analysis. The retroperitoneal fat pad and liver were weighed, and livers were kept at −80°C.

### Bioassays

Plasma, liver extracts and glycerol standards (Sigma-Aldrich, MO, United States) were incubated with triacylglycerol reagent (Roche Diagnostics, Basel, Switzerland) using an in-house assay as we previously described (Chen et al., 2018c). Plasma non-esterified free fatty acids (NEFA) were measured using a commercial NEFA kit (WAKO, Osaka, Japan) following the manufacturer’s instructions (Chen et al., 2018c). Total liver lipid was measured by semi-quantitative oil red O (ORO) assay, as we previously described (Lo et al., 2011). Briefly, the ORO stock solution 0.25% (wt/vol) was freshly diluted in 10% dextran at 6:4 (ORO: dextran); then, 30–50mg liver was homogenised and incubated in the ORO working solution for 1h. After washing with 60% isopropanol to remove excess dye, the dye incorporated into lipid was extracted in 99% isopropanol. Absorbance at 520nm was measured alongside an ORO standard curve.

### Real-Time PCR

Gene expression was measured using our previously published protocol (Li et al., 2019) according to the MIQE guidelines (Bustin et al., 2009). Briefly, total mRNA was extracted from liver tissue with TriZol reagent following the manufacturer’s instructions (Life Technologies, CA, United States) and used as a template to generate the first-strand cDNA using M-MLV Reverse Transcriptase, RNase H, Point Mutant Kit (Promega, WI, United States). Gene expression was quantified with manufacturer pre-optimised and validated TaqMan^®^ primers and probes pre-optimised by the manufacture (Thermo Fisher, CA, United States) and standardised to 18s RNA (Li et al., 2019). The expression was calculated using 2^−∆∆Ct^ and the Chow+sham group was assigned the calibrator against which all other results were expressed as fold changes.

### Western Blotting

Western blotting was performed for fatty acid synthase (FASN). The liver was homogenised using cell lysis buffer (Hepes 20mm, EGTA 1mm, Mannitol 210mm and Sucrose 70mm) for the whole protein with protease inhibitor (Thermo Fisher, United States, Cat. A32963, dissolve fresh in 50ml of lysis buffer). Protein samples (40μg) were separated on 4–15% Criterion TGX Precast Midi Protein Gel, 26 well (Bio-Rad, United States, Cat. 5671085) and then transferred to PVDF membranes with Trans-Blot Turbo Transfer System (Trans-blot Turbo Midi 0.2μm PVDF Transfer pack; Bio-Rad, United States, Cat. 1704157), which were blocked with non-fat milk powder and incubated with the primary antibodies FASN (1:2,000, Cat#3180S, Cell Signalling) and β-action (1:1,000, Cat#AHP2417, Bio-Rad) overnight and then secondary antibodies (1:5,000, goat anti-rabbit IgG horseradish peroxidase-conjugated secondary antibodies, Cat#STAR124P, BIO-RAD) for 1h. Protein expression was detected by SuperSignal West Pico Chemiluminescent substrate (Thermo, MA, United States) by exposure of the membrane in Chemidoc MP (Bio-Rad, California, United States). Protein band density was measured using ImageJ software (National Institute of Health, Bethesda, Maryland, United States), with the results expressed as a ratio of the individual marker intensity relative to β-actin band intensity.

### Statistical Methods

The results are expressed as mean±standard error of the mean (SEM) and analysed by two-way ANOVA followed by Fisher’s least significant difference (LSD) *post-hoc* tests (GraphPad Prism 9, GraphPad, CA, United States). A conditional *t*-test was performed between the control (Chow+sham or HFD+sham) and interventional groups. *p*<0.05 was considered statistically significant.

## Results

### Adiposity and Lipid Profile

All the groups had a similar body weight at baseline (Table 1). After 16weeks of HFD consumption, the mice consuming a HFD were heavier than chow-fed mice receiving the same treatment (*p*<0.05 HFD+sham vs. Chow+sham group, Table 1). Nicotine-free e-vapour reduced body weight in chow-fed mice only (*p*<0.05, Chow+e-cig0 vs. Chow+sham). There was a trend of e-vapour exposure to reduce daily caloric intake independent of nicotine (Table 1), where only Chow+e-cig0 group ate significantly less than the Chow+sham group (*p*<0.05 Table 1), consistent with their smaller body weight. Liver weight was not changed by HFD but was reduced by e-vapour exposure (*p*<0.01 overall e-vapour exposure effect; *p*<0.05 Chow+e-cig18 and Chow+e-cig0 vs. Chow+sham, HFD+sham vs. HFD+e-cig0, Table 1). HFD consumption nearly doubled the amount of retroperitoneal and epididymal fat masses (for both fat pads *p*<0.01, Chow+sham vs. HFD+sham, HFD+e-cig18 vs. Chow+e-cig18; epididymal fat *p*<0.01 HFD+e-cig0 vs. Chow+e-cig0, Table 1). In chow-fed mice, e-vapour exposure also reduced retroperitoneal fat mass regardless of nicotine content (*p*<0.05 vs. Chow-sham), with a similar trend in epididymal fat albeit no statistical significance. However, in HFD-fed mice, while retroperitoneal was reduced by exposure to nicotine-free e-vapour (*p*<0.05 HFD+e-cig0 vs. HFD+sham), epididymal fat was not significantly changed by e-vapour exposure (Table 1).

**Table 1:** Metabolic parameters.

	Chow+sham	Chow+e-cig18	Chow+e-cig0	HFD+sham	HFD+e-cig18	HFD+e-cig0	Main effect (*p*)
Body weight at week 0 (g)	20.3 ± 0.26	20.5 ± 0.29	20.2 ± 0.20	20.4 ± 0.31	20.2 ± 0.43	20.2 ± 0.25	
Body weight at week 16 (g)	29.0 ± 0.24	27.4 ± 0.6	26.6 ± 0.5^*^	30.3 ± 0.60*^t^*	29.2 ± 0.81	28.2 ± 0.46	diet<0.01
							e-vapour<0.01
Caloric intake (kJ/mouse/day)	58.0 ± 0.50	52.7 ± 3.31	49.3 ± 3.88^*^	54.6 ± 2.21	48.7 ± 3.24	49.7 ± 2.39	
Liver (g)	1.26 ± 0.04	1.14 ± 0.03^*^	1.12 ± 0.03^*^	1.23 ± 0.05	1.20 ± 0.03	1.11 ± 0.04^#^	e-vapour<0.01
Liver %	4.57 ± 0.14	4.37 ± 0.14	4.38 ± 0.09	4.32 ± 0.14	4.18 ± 0.06	4.02 ± 0.10^‡^	diet<0.01
Retroperitoneal white fat (g)	0.19 ± 0.02	0.14 ± 0.02*^t^*	0.13 ± 0.01*^t^*	0.35 ± 0.05^**^	0.31 ± 0.04^††^	0.22 ± 0.02^#^	diet<0.01
							e-vapour<0.05
Retroperitoneal white fat (%)	0.68 ± 0.07	0.45 ± 0.08*^t^*	0.51 ± 0.06*^t^*	1.17 ± 0.16^*^	1.06 ± 0.13^††^	0.81 ± 0.06^#^	diet<0.01
							e-vapour=0.05
Epididymal white fat (g)	0.50 ± 0.03	0.43 ± 0.05	0.40 ± 0.02	0.92 ± 0.08^**^	0.89 ± 0.10^††^	0.82 ± 0.05^‡‡^	diet<0.01
Epididymal white fat (%)	1.82 ± 0.11	1.64 ± 0.16	1.55 ± 0.07	3.11 ± 0.21^**^	3.32 ± 0.24^††^	2.98 ± 0.16^‡‡^	diet<0.01
AUC (mm·min)	1,326 ± 54	1,206 ± 70*^t^*	1,274 ± 38	1,481 ± 62^*^	1,252 ± 42^##^	1,390 ± 33	diet<0.05
							e-vapour<0.01
Plamsa NEFA (nmol)^η^	3.71 ± 0.38	4.75 ± 0.36^*^	4.65 ± 0.23	4.41 ± 0.32^*^	4.60 ± 0.11	5.33 ± 0.48^#^	e-vapour<0.05
Plasma tryglycerides (mg/ml)^η^	1.87 ± 0.11	2.05 ± 0.14*^t^*	2.38 ± 0.19*^t^*	2.75 ± 0.28^**^	2.09 ± 0.22^#^	2.43 ± 0.18	
Liver Oil red O (mg/g liver)^η^	45.6 ± 9.25	76.2 ± 10.6^*^	79.1 ± 14.4^*^	87.9 ± 8.75^*^	82.5 ± 7.92	80.3 ± 7.08	

*The results are expressed as mean±SEM, n=15. Data were analysed by two-way ANOVA followed by Fisher’s least significant difference (LSD) post-hoc tests. A conditional t-test for non-overlap of value distributions was performed between the control (Chow+sham or HFD+sham) and interventional groups*.

η *n=7–8*.

* *p<0.05;*

** *p<0.01* vs. *Chow+Sham*;

t *p<0.05 by conditional t-test* vs. *Chow+Sham*.

# *p<0.05*; ^##^p<0.01 vs. *HFD+Sham*.

†† *p<0.01* vs. *Chow+e-cig18*.

‡ *p<0.05*; ^††^p<0.01 vs. *Chow−e-cig0*.

*AUC, area under the curve of blood glucose concentrations during an intraperitoneal glucose tolerance test; NEFA, non-esterified fatty acids*.

High-fat diet consumption increased blood NEFA levels as expected (*p*<0.05, HFD+sham vs. Chow+sham group). There was an overall effect of e-vapour exposure to increase NEFA level in both chow and HFD-fed mice (*p*<0.05); while significance was only seen between Chow+sham and Chow+e-cig18 groups (*p*<0.05), and between HFD+sham and HFD+e-cig0 groups (*p*<0.05). HFD consumption also increased serum triglycerides levels (*p*<0.01, HFD+sham vs. Chow+sham group, Table 1), which was reduced by exposure to nicotine-containing e-vapour (*p*<0.05, HFD+e-cig18 vs. HFD+sham, Table 1). However, in chow-fed mice, blood triglycerides were increased by e-vapour exposure independent of nicotine (*p*<0.05 Chow+e-cig18 and Chow+e-cig0 vs. Chow+sham by conditional *t*-tests). Interestingly, the triglyceride concentration in the liver followed a similar pattern to triglycerides in the blood (*p*<0.05, Chow+sham vs. Chow+e-cig18, Chow+e-cig0 and HFD+sham, Table 1).

**Figure 1:**
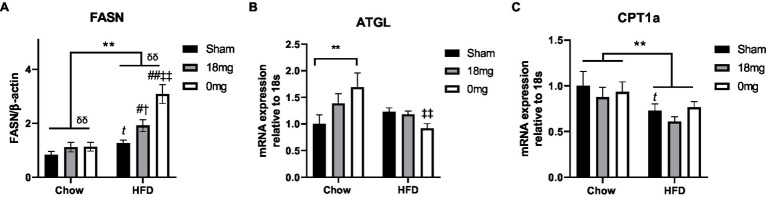
Lipid metabolic markers in the liver. Protein level of FASN **(A)**, mRNA expression of ATGL **(B)** and CPT1a **(C)** in mice fed a HFD with or without the exposure to e-vapour. The results are expressed as mean±SEM, *n*=5–8. Data were analysed by two-way ANOVA followed by Fisher’s least significant difference (LSD) *post-hoc* tests. A conditional *t*-test for non-overlap of value distributions was performed between the control (Chow+sham or HFD+sham) and interventional groups. ^**^p<0.01 diet effect, ^δδ^*p*<0.01 e-vapour exposure effect; *^t^p*<0.05 vs. Chow+Sham by conditional *t*-test, ^#^*p*<0.05, ^##^*p*<0.01 vs. HFD+Sham, ^†^*p*<0.05 vs. Chow+e-cig18mg and ^‡‡^*p*<0.01 vs. Chow+e-cig0. ATGL, adipose triglyceride lipase; CPT, carnitine palmitoyltransferase; and FASN, fatty acid synthase.

The *de novo* lipogenesis marker fatty acid synthase (FASN) was increased by HFD consumption and e-vapour exposure (*p*<0.05, HFD+sham vs. Chow+sham, HFD+e-cig18 vs. HFD+sham and Chow+e-cig18), with the effect more robust in the HFD+e-cig0 group (*p*<0.01 vs. HFD+sham and Chow+e-cig0, Figure 1A). The lipolysis marker adipose triglyceride lipase (ATGL) was upregulated by nicotine-free e-vapour in chow-fed mice (*p*<0.01, Chow+e-cig0 vs. Chow+sham, Figure 1B) but was suppressed in HFD-fed mice with the same treatment (*p*<0.01, HFD+e-cig0 vs. HFD+sham, Figure 1B). The rate-limiting enzyme for lipid β-oxidation, carnitine palmitoyltransferase (CPT)1a, was suppressed by HFD consumption (*p*<0.01, overall diet effect; *p*<0.05, HFD+sham vs. Chow+sham, Figure 1C), without any influence from e-vapour exposure.

### Glucose Metabolism

**Figure 2:**
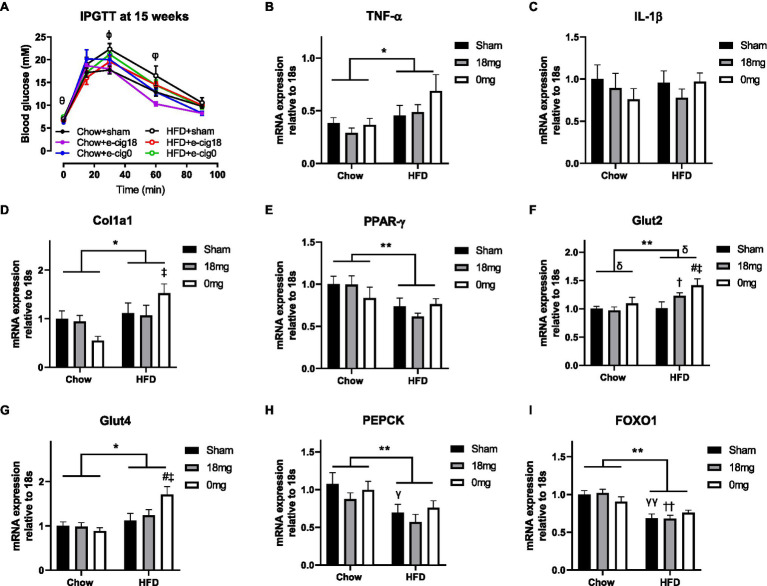
Glucose level during intraperitoneal glucose tolerance test (IPGTT, **A**) and mRNA expression of markers of inflammation [TNF-α **(B)**, IL-1β **(C)**], fibrosis (Col1a1, **D**), insulin sensing (PPARγ, **E**), glucose transporter [Glut2 **(F)**, Glut4 **(G)**] and gluconeogenesis [PEPCK **(H)**, FOXO1 **(I)**] in the liver. The results are expressed as mean±SEM, *n*=5–8. Data were analysed by two-way ANOVA followed by Fisher’s least significant difference (LSD) *post-hoc* tests. ^θ^*p*<0.05 Chow+e-cig0 vs. HFD+e-cig0; ^ϕ^*p*<0.01 Chow+sham vs. HFD+sham; ^φ^*p*<0.05 Chow+sham vs. HFD+sham; Chow+e-cig18 vs. HFD+e-cig18; ^*^p<0.05, ^**^p<0.01 diet effect, ^δ^*p*<0.05 e-vapour exposure effect; ^γ^*p*<0.05, ^γγ^*p*<0.01 vs. Chow+sham; and ^#^*p*<0.05 vs. HFD+Sham, ^†^*p*<0.05; ^††^*p*<0.01 vs. Chow+e-cig18 and ^‡^*p*<0.05 vs. Chow+e-cig0. Col1a1, collagen 1a1; FOXO1, Forkhead box protein O1; Glut, glucose transporter; PEPCK, phosphoenolpyruvate carboxykinase; and PPAR, Peroxisome proliferator-activated receptors.

During the glucose tolerance test, the glucose levels in the HFD+sham group were significantly higher than the Chow+sham group at 30 and 60min post-glucose injection (*p*<0.05, Figure 2A). The HFD+cig18 group only had a higher glucose level than its chow-fed counterpart at 60min (*p*<0.05 vs. Chow+e-cig18), and the HFD+cig0 group had a higher glucose level than its chow-fed counterpart before glucose injection at Time 0 (*p*<0.05 vs. Chow+e-cig0, Figure 2A). Glucose intolerance in HFD+sham mice was confirmed by a greater AUC value for the glucose tolerance test (*p*<0.05, HFD+sham vs. Chow+sham group, Table 1), which was reduced by exposure to nicotine-containing e-vapour (*p*<0.01, HFD+e-cig18 vs. HFD+sham). A similar effect was found in chow-fed mice (*p*<0.05 by conditional *t*-test, Chow+e-cig18 vs. Chow+sham).

We examined markers related to insulin sensitivity and glucose metabolism. The inflammatory marker TNF-α was increased by HFD consumption (*p*<0.05 diet effect, Figure 2B), but no change was observed for IL-1β (Figure 2C), suggesting activation of macrophages but not the inflammasome. The total intrahepatic expression of collagen 1 (Col1a) was also increased by HFD, as expected (*p*<0.05, Figure 2D), with significance observed between the Chow+e-cig0 and HFD+e-cig0 groups (*p*<0.05). HFD suppressed the expression of the insulin sensing marker PPARγ (*p*<0.01, Figure 2E), which was not affected by e-vapour exposure. There was an overall effect of diet to increase the intrahepatic expression of both Glut 2 (*p*<0.01, Figure 2F) and Glut 4 (*p*<0.05, Figure 2G), and e-vapour exposure increased Glut 2 expression (*p*<0.05, HFD+e-cig18 vs. HFD+sham, HFD+e-cig0 vs. HFD+sham). Overall, the changes in most of the above-mentioned markers were more marked in HFD+e-cig0 group, suggesting potential harm from the combination of HFD and exposure to nicotine-free e-vapour. Two gluconeogenesis markers, phosphoenolpyruvate carboxykinase (PEPCK) and Forkhead box protein O1 (FOXO1), were both suppressed by HFD consumption (*p*<0.01 overall diet effect, Figures 2H,I) with no influence from e-vapour exposure.

## Discussion

While there is no firm conclusion on whether e-cigarette use increases the risk of type 2 diabetes (Górna et al., 2020), our controlled animal model showed that sub-chronic exposure to a low dose of e-cigarette vapour does not impair glucose tolerance. On the contrary, in HFD-fed mice, nicotine-containing e-vapour improved glucose clearance, while nicotine-free e-vapour reduced retroperitoneal fat mass. However, while e-vapour exposure reduced fat mass in both chow and HFD-fed mice, there was a differentiated impact of e-vapour exposure on the lipid profile. In chow-fed mice, lipids in blood and liver were increased by e-vapour exposure independent of nicotine. In HFD-fed mice, blood NEFA and triglycerides were increased by nicotine-free e-vapour, while triglycerides were reduced by nicotine-containing e-vapour, with no impact on the liver triglycerides.

We used a HFD with a lower fat concentration (43%) than others have used (>60%; Espinoza-Derout et al., 2019; Hasan et al., 2019). However, energy from the dietary fat in this study is still higher than the average intake of adult Australian males, and >22% higher than the upper limit of the accepted macronutrient distribution range (Grech et al., 2018). Different from our previous observations of significant weight gain in C57BL/6 mice using the same diet (Glastras et al., 2016; Chen et al., 2018b,c; Komalla et al., 2020), Balb/c mice had less than 5% weight gain, yet with nearly doubled retroperitoneal and epididymal fat masses. This is consistent with the strain susceptibility to HFD-induced obesity (Montgomery et al., 2013). In line with increased adiposity, blood levels of NEFA and triglycerides were also increased, suggesting the key role of fat mass in determining metabolic disorders. Therefore, 16weeks of HFD consumption is sufficient to induce metabolic disorders. E-vapour exposure reduced retroperitoneal fat mass regardless of diet. This is somewhat similar to a previous study using cigarette smoke exposure with a similar nicotine level (Chen et al., 2007). Although one may consider this to be due to an effect of nicotine, retroperitoneal fat mass reduction in HFD-fed mice was more marked with nicotine-free e-vapour than the nicotine-containing one, with a similar trend observed in epididymal fat. This fat loss somewhat mirrors the suppressed caloric intake by e-vapours exposure which was independent of nicotine. This result suggests that both suppressed appetite and fat loss effect is most likely due to other chemicals in the e-vapour other than nicotine. Here, we cannot rule out the possibility of other additives [e.g. pyrazines (Clineschmidt et al., 1977)] in the e-vapour that may activate the reward pathway to suppress appetite, which requires future studies to identify.

There has been a dramatic increase in non-alcoholic fatty liver disease in recent years, due to increased intake of diets rich in simple carbohydrates and lipids that activate *de novo* lipid synthesis (Loomba et al., 2021). In this study, there was >60% increase in liver lipid storage in HFD-fed mice (HFD+sham). Very likely contributing to increased lipid deposition, the enzyme for lipid synthesis FASN was upregulated while the regulator of lipid β-oxidation CPT-1a was downregulated, whereas the lipolytic enzyme ATGL was not changed. In addition, hepatic insulin resistance can reduce glucose conversion to glycogen and re-direct it to *de novo* lipid synthesis (Loomba et al., 2021). Indeed, the insulin sensing marker PPARγ was downregulated in HFD+sham mice, supporting this mechanism. HFD-fed mice had a significant increase in FASN from nicotine-containing e-vapour, and more so from nicotine-free e-vapour exposure. However, this change negatively correlated with triglyceride concentrations in the liver. As invoking the classical lipid metabolic regulators does not explain such changes from e-vapour exposure in HFD-fed mice, there may be an unknown mechanism that requires further investigation. Conversely, although nicotine has been shown to promote lipid accumulation in the liver (Sinha-Hikim et al., 2017; Chen et al., 2018d), here in chow-fed mice increased liver triglycerides seems to be independent of nicotine. It is more likely that the glycerine base of e-vapour can enter the bloodstream from the respiratory tract without restriction (Eissenberg and Maziak, 2020), which may be a contributor to increased blood and liver lipids in chow-fed mice exposed to e-vapour. A study in humans found that the addition of glycerine to a high-fat meal increased plasma free fatty acid level, possibly *via* chylomicron synthesis (Nicolaïew et al., 1995). While there is no direct evidence to support this speculation, a toxicity study did suggest intact humectant vegetable glycerine after heating (Li et al., 2021). Future studies can investigate this possibility. Nevertheless, lipolysis regulator ATGL was only upregulated by e-vapour exposure in chow-fed mice, which may also be an adaptive response to increased lipid content in the liver that is not successful in reversing the condition. This adaption can be impaired by long-term HFD consumption; thus, ATGL was not increased in HFD-fed mice with the same e-vapour exposure. In summary, e-vapour exposure may increase the risk of fatty liver disorder regardless of nicotine, which does not exacerbate lipid deposition due to HFD consumption.

The liver regulates peripheral glucose metabolism. Local inflammation due to residential macrophages plays a key role in liver insulin resistance due to HFD diet consumption (Lo et al., 2011), exemplified by increased TNF-α and synchronously reduced PPARγ expression in HFD-fed mice in this study. It is believed that nicotine is anti-inflammatory (Kalra et al., 2004; Cohen et al., 2007), which was not reflected in the HFD+e-cig18 and HFD+e-cig0 mice, potentially due to the low dose of nicotine in this study. Liver fibrosis is directly linked to local inflammation and ectopic lipid accumulation. Here, collagen was increased only by HFD consumption and not by e-vapour exposure.

Forkhead box protein O1 and its promoter PEPCK regulate gluconeogenesis, raising blood glucose levels. They are upregulated during dietary obesity and type 2 diabetes, resulting in hyperglycaemia and glucose intolerance (Nakae et al., 2001; Seth et al., 2013). As a result, suppressing FOXO1 signalling can protect mice from HFD-induced insulin resistance by increasing insulin sensing regulator PPARγ (Kim et al., 2009). However, in this model, both PEPCK and FOXO1 were suppressed by HFD. We hypothesise it may be another adaptative response. Our results of improved glucose tolerance in the Chow+e-cig18 and HFD+e-cig18 mice are similar to a previous study using a low level e-vapour exposure and chronic nicotine administration (Thompson et al., 1990; Vu et al., 2014; Orimoloye et al., 2019). Moreover, PEPCK has also been shown to be suppressed by chronic nicotine administration (Vu et al., 2014). Previously, cold e-fluid which has a different composition to e-cigarette vapour generated by an e-cigarette device, was intraperitoneally injected to study glycaemic control (El Golli et al., 2016). To our knowledge, no other study has used a setting similar to ours (i.e. using e-vapour with/without nicotine in combination with a HFD), with which to directly compare the finding. Nevertheless, the improved glucose tolerance by nicotine-containing e-vapour is similar to the effect of tobacco cigarette smoke at a does equivalent to light smokers (Chen et al., 2007). The comparison with the groups exposed to nicotine-free e-vapour (HFD+e-cig0) further suggests that the improvement of glucose tolerance is most likely to be nicotine driven, although our studies have not defined the mechanistic basis for this improvement.

We demonstrated increased glucose transporters in HFD-fed mice with e-vapour exposure, especially nicotine-free e-vapour. With downregulated gluconeogenesis markers, this may partially explain why glucose tolerance was improved in mice exposed to e-vapour. However, the glycaemic control effect was still more potent with nicotine-containing e-vapour, consistent with the effect of pure nicotine to increase glucose uptake in the literature (Liu et al., 2003; Vu et al., 2014). However, glucose may not be converted to triglycerides for storage. How the additional glucose was disposed of needs further investigation.

We need to acknowledge several limitations in this study. Firstly, we only used a low dose of e-vapour exposure, which is equivalent to light smokers. Higher doses of e-vapour may introduce more toxic chemicals, potentially having different effects on lipid and glucose metabolism. Secondly, we exposed the mice to e-vapour for 6weeks, which is a relatively short duration compared with human usage. Some improvement in glucose or adiposity may be a ‘honeymoon’ adaptative effect, considering the unfavourable changes in FASN, PPARγ and glucose transporters in the liver. Thirdly, we used Balb/c mice, which is not an obese-prone strain and the ability of the liver to uptake lipids is also lower than other strains (Montgomery et al., 2013). As such, the adverse or beneficial effects on nutrient metabolism of e-vapour exposure need to be interpreted with caution. Lastly, this short communication only reports the initial finding in the males to raise the attention of the field in the setting of HFD combined by e-vaping. We would expect similar changes in the females, as there seems to be no sex difference in the metabolic disturbance in response to environmental insults (Nguyen et al., 2015; Saad et al., 2018). Future studies can follow up on the mechanisms, such as the involvement of the gut microbiome, and confirm the changes in the females.

## Conclusion

In this study, e-vapour exposure reduced fat mass in both chow and HFD-fed mice but showed a differential effect on lipid profile with different diets. In mice fed a balanced diet, it tends to increase lipid levels in both blood and liver. However, in mice fed a diet rich in lipid and simple carbohydrates, it tends to reduce triglycerides but increase NEFA levels in the blood. In addition, low-dose nicotine inhalation seems to benefit glucose tolerance in HFD-fed mice; however, this should not be translated to humans who have more lifestyle confounders than laboratory animals.

## Data Availability Statement

The original contributions presented in the study are included in the article/supplementary material, and further inquiries can be directed to the corresponding author.

## Ethics Statement

The animal study was reviewed and approved by the Animal Ethics and Care Committee at Northern Sydney Local Health District.

## Author Contributions

HC, SS, and BO designed the study. HC, GL, YC, and HZ performed the experiments, collected the data, and analysed the results. HC prepared the figures and tables and wrote the first draft. All authors contributed to the writing of the manuscript, reviewed the final manuscript and have read and agreed to the published version of the manuscript.

## Funding

This work was supported by a project grant from Australian National Health and Medical Research Council (AP1158186) and University of Technology Sydney.

## Conflict of Interest

The authors declare that the research was conducted in the absence of any commercial or financial relationships that could be construed as a potential conflict of interest.

## Publisher’s Note

All claims expressed in this article are solely those of the authors and do not necessarily represent those of their affiliated organizations, or those of the publisher, the editors and the reviewers. Any product that may be evaluated in this article, or claim that may be made by its manufacturer, is not guaranteed or endorsed by the publisher.

## Abbreviations

ATGL: adipose triglyceride lipase
AUC: area under the curve of glucose change during the intraperitoneal glucose tolerance test
CPT: carnitine palmitoyltransferase
FASN: fatty acid synthase
FOXO1: Forkhead box protein O1
HFD: high-fat diet
IPGTT: intraperitoneal glucose tolerance test
NEFA: non-esterified fatty acids
ORO: oil red O
Glut: glucose transporter
PEPCK: phosphoenolpyruvate carboxykinase
PPAR: peroxisome proliferator-activated receptor
SEM: standard error of the mean
